# Eco-Friendly Green Synthesis and Characterization of Silver Nanoparticles by *Scutellaria multicaulis* Leaf Extract and Its Biological Activities

**DOI:** 10.3390/ph16070992

**Published:** 2023-07-11

**Authors:** Zahra Gharari, Parichehr Hanachi, Hanie Sadeghinia, Tony R. Walker

**Affiliations:** 1 Department of Biotechnology, Faculty of Biological Sciences, Alzahra University, Tehran 1993893973, Iran; sadeghinia.h@yahoo.com; 2 School for Resource and Environmental Studies, Dalhousie University, Halifax, NS B3H 4R2, Canada; trwalker@dal.ca

**Keywords:** apoptotic, cytotoxicity, green synthesis, nanostructures, *Scutellaria multicaulis*, silver

## Abstract

*Scutellaria multicaulis* is a medicinal plant indigenous to Iran, Afghanistan, and Pakistan. It has been widely used as a prominent herb in traditional medicine for thousands of years. This plant is reported to contain baicalein, wogonin, and chrysin flavonoids, which are a significant group of chemical ingredients which can cure different diseases, such as breast cancer. *S. multicaulis* leaf extract was used for the bioreduction of silver nanoparticles (SmL-Ag-NPs), and their phytochemical contents and antioxidant, antibacterial, anti-proliferative, and apoptotic activity were evaluated. Optimal physicochemical properties of SmL-Ag-NPs were obtained by mixing 5% of leaf extract and 2 mM of aqueous AgNO_3_ solution and confirmed by characterization studies including UV–visible spectrophotometry, Field Emission Scanning Electron Microscope (FE-SEM), Energy Dispersive X-ray (EDX), Dynamic Light Scattering (DLS), zeta potential, Thermogravimetric analysis (TGA), Surface-enhanced Raman spectroscopy (SERS), X-ray crystallography (XRD), and Fourier transform infrared (FTIR) Spectroscopy. SmL-Ag-NPs exhibited a higher content of total phenol and total flavonoid and potential antioxidant activity. SmL-Ag-NPs also demonstrated dose-dependent cytotoxicity against MDA-MB231 cell multiplication with an IC_50_ value of 37.62 μg/mL through inducing cell apoptosis. Results show that SmL-Ag-NPs is effective at inhibiting the proliferation of MDA-MB231 cells compared to tamoxifen. This demonstrates that SmL-Ag-NPs could be a bio-friendly and safe strategy to develop new cancer therapies with a reduction in the adverse effects of chemotherapy in the near future.

## 1. Introduction

Despite significant breakthroughs in disease prevention, control, and treatment, cancer is still a major public health problem around the globe. Currently, chemotherapy is the most common primary treatment for cancer and is used to treat most cancers. However, due to the resistance of tumor cells to the chemotherapy agents, it eventually results in therapeutic failure and death [1]. Genetic instability, high rates of mutation, and rapid changes in the genetics of cancerous cells makes them resistant to drugs [1]. Therefore, discovering a broad and effective treatment strategy to reduce or reverse multi-drug resistance to cancer is urgently required. Nanotechnology has brought a revolution in cancer diagnosis, detection, and treatment [2].

Over the past decades, anti-angiogenesis and the anti-cancer activities of AgNPs have steadily received much attention in medicine [3]. Notably, many in vitro studies have indicated the considerable potential of silver nanoparticles for cancer treatment due to their proven antitumor effect [3]. Based on the reducing agents involved in reducing metallic ions, various preparation techniques, such as chemical reduction, physical reduction, photochemical methods, and biological approaches, have been reported. Each approach has its crucial strengths and limitations [4]. However, compared to overall physical and chemical strategies, biogenic nanoparticles are advantageous as their synthesis does not involve hazardous chemicals for reduction and stabilization. Biological synthesis for micro/nanoparticles is particularly desirable because the tactic is environmentally benign, energy-efficient, cost-effective, sustainable, and readily used in reliable electrical interconnects and integrated circuits [5]. Hence, there is an increasing tendency to use a biological approach called green synthesis of nanoparticles. Green nanotechnology using plants and microbes has attracted much attention in materials science and the medicinal, pharmaceutical, and textile industries [6]. Nanoparticle biosynthesis through various macro–microscopic organisms such as plants, fungi, algae, and bacteria as reducing or capping agents has been carried out and discussed by a number of different studies [7]. Several biological constituents, such as terpenoids, flavonoids, carboxylic acids, ketones, amides, aldehydes, and ascorbic acids, have been adopted to take part in the synthesis of reduced and stabilized forms of silver nanoparticles [7]. Owing to their attractive and unique nano-related properties, outstanding bactericidal activity, and enhanced antitumor activity, silver nanoparticles (AgNPs) have been one of the most extensively used nanomaterials in the biomedicine, household utensils, and food industries [8].

*Scutellaria multicaulis* Boiss. belongs to the Lamiaceae family. The native range of this species is from Iran to the western Himalayas. Various plant species in the genus Scutellaria have been used as a local remedy to treat cancer, hepatic disorders, anxiety, cirrhosis, jaundice, infection, and hepatitis problems [9]. Studies show that the pharmacological actions of Scutellaria are related to the presence of its active principles, flavonoids, mainly baicalein, wogonin and its glycoside forms, baicalin, and wogonoside, as well as terpenes [10]. In several in vitro techniques, bioactive phytochemicals in some species of Scutellaria exhibited potent anticancer activity against some human cancers, antibacterial effects on human pathogenic bacteria, and antioxidant and chelating abilities [11,12]. In several in vitro techniques, the bioactive phytochemicals of some species of Scutellaria, including phenylpropanoids, phenylethanoids, flavones, and essential oils, exhibited potent anticancer activity against some of the human cancers, antibacterial effects on human pathogenic bacteria, and antioxidant and chelating abilities in various studies [7]. Due to the remarkable and broad-ranging biological activities of Scutellaria species as well as the attractive physiochemical properties of AgNPs, the synthesis of silver-based nanoparticles using various parts of Scutellaria plants such as root extract of *Scutellaria baicalensis* [13], stem extracts of *Scutellaria multicaulis* [7], *Scutellaria Iscandaria* extract [14,15], and aqueous extract of *Scutellaria barbata* [16] has attracted tremendous interest for application in the biomedical field in the past years. 

Breast cancer is a significant public health problem, accounting for approximately 10 million deaths worldwide in 2020. According to Cancer statistics, 287,850 new cases of invasive breast cancer, 51,400 cases of ductal carcinoma in situ (DCIS), and 43,250 cancer deaths were projected to occur worldwide in 2022 [17]. As mentioned above, the present study investigated the anticancer potency of green synthesized silver nanostructures using *S. multicaulis* leaf extract against the growth of malignant MDA-MB-231 cell line, their antibacterial activity against bacterial pathogens, antioxidant ability, and phytochemical composition. To our knowledge, this is the first report about the anticancer activity of biosynthesized silver nanostructures using *S. multicaulis* leaf extract.

## 2. Results and Discussion 

### 2.1. Biosynthesis and Characterization of SmL-Ag-NPs Using UV-Visible Spectroscopy Analysis

**Figure 1:**
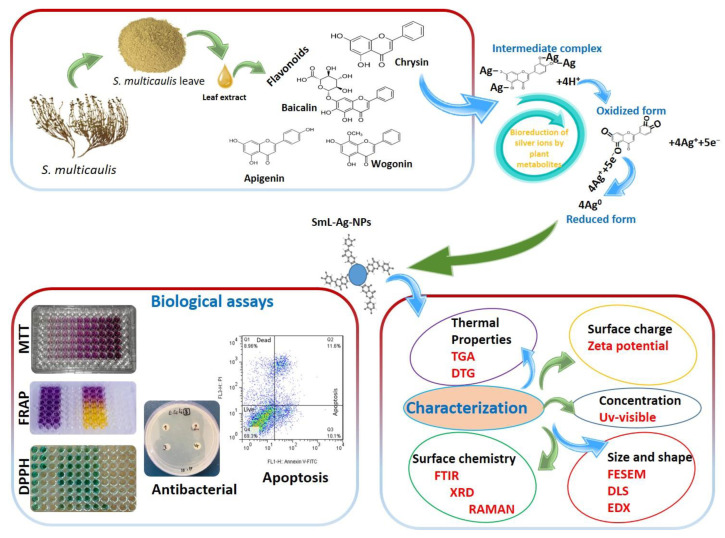
A representative schematic of the green synthesis, characterization, and biological tests of silver nanoparticles using *Scutellaria multicaulis* leaf extract.

**Figure 2:**
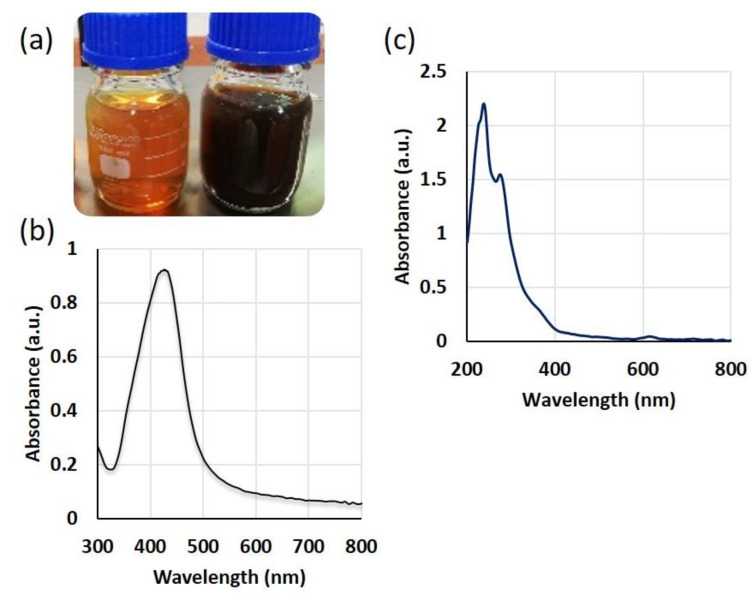
(**a**) Visual observation of the reduction of silver ions to SmL-Ag-NPs; UV-Visible spectroscopy analysis of (**b**) SmL-Ag-NPs and (**c**) plant extract.

SmL-Ag-NPs were successfully biosynthesized using different volumes of *S. multicaulis* leaf extract (1 mL, 2.5 mL, and 5 mL) and different volumes (99, 97.5, and 95 mL) and concentrations (0.5, 1, and 2 mM) of aqueous silver nitrate solution. Figure 1 shows a schematic procedure of SmL-Ag-NPs using *S. multicaulis* leaf extract, its characterization, and biological tests. *S. multicaulis* leaf extract-mediated SmL-Ag-NPs synthesis is generally started by mixing the extract with different concentrations of AgNO_3_ salt solution (0.5, 1, and 2 mM) at room temperature. The formation of SmL-Ag-NPs is fast and initially identified based on a visual color change from colorless to brown within one hour and finally to dark brown after overnight incubation, indicating the formation of SmL-Ag-NPs (Figure 2a). The nucleation of the reduced metal ions from monovalent to divalent oxidation states occurs initially. This step is immediately followed by the periodic development of neighboring particles to form bigger nanoparticles via noncovalent interactions, which are thermodynamically stable, while extra metal ion reduction continues. At the final step of the synthesis, the *S. multicaulis* leaf extract stabilizes the SmL-Ag-NPs, resulting in a more energetically stable morphology (Figure 1). A variety of bioactive chemicals, including flavonoids and phenols, are involved in SmL-Ag-NPs formation. The functional components of these bioactive compounds operate as reducing and stabilizing agents for SmL-Ag-NPs biosynthesis. Figure 1 shows the mechanism of SmL-Ag-NPs formation by using *S. multicaulis* leaf extract. UV-Vis spectroscopy has been used to prove the synthesis of SmL-Ag-NPs. UV spectra of surface plasmon resonance peaks of SmL-Ag-NPs were observed at 435.89 nm, similar to UV–Vis spectra Sm-AgNPs biosynthesized using *S. multicaulis* stem extract, suggesting the formation of well-stabilized SmL-Ag-NPs (Figure 2b) [7]. UV–Visible spectra of *S. multicaulis* leaf extract are presented in Figure 2c.

### 2.2. Field Emission Scanning Electron Microscope/Energy Dispersive X-ray (FE-SEM/EDX) Analysis of SmL-Ag-NPs

**Figure 3:**
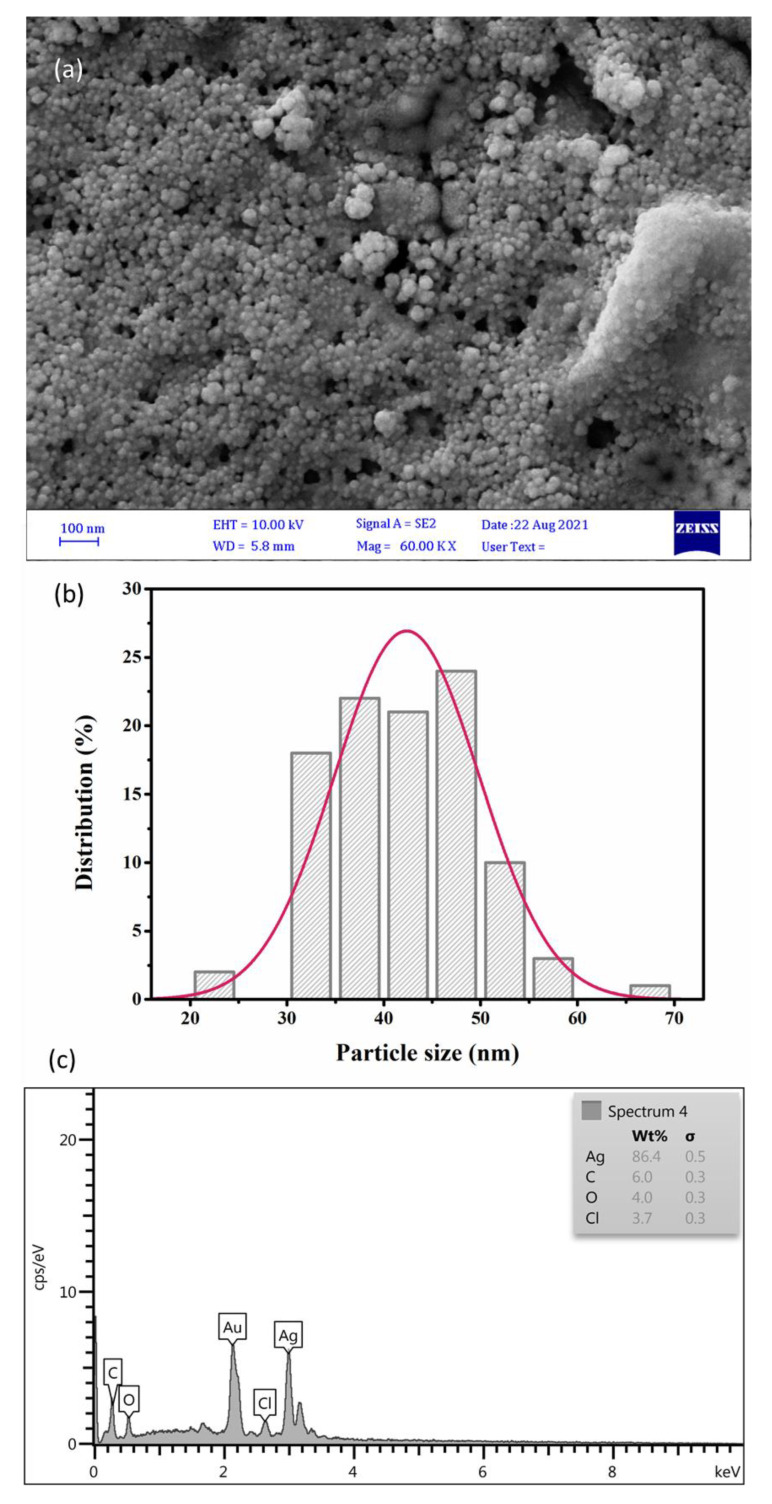
(**a**) FE-SEM micrograph of silver nanoparticles synthesized using *S. multicaulis* leaf extract; (**b**) Histogram of the particle size distribution; and (**c**) EDX spectrum of SmL-Ag-NPs. FE-SEM: Field emission scanning electron microscopy; EDX: Energy Dispersive X-ray Analysis.

FE-SEM/EDX analysis as an advanced technology was used to visualize the morphological and topographical information on the surface of fabricated SmL-Ag-NPs (Figure 3). FE-SEM images confirmed that SmL-Ag-NPs were mainly spherical and oval and mono-dispersed in the particle size ranging from 31 to 58 nm and an average size of 42.5 nm (Figure 3a). Figure 3b shows the FE-SEM histogram of the particle size distribution of SmL-Ag-NPs, which is plotted by analyzing several frames of the same FE-SEM images. The results agreed with the similar finding reported by Gharari et al. (2022), where the FE-SEM studies show that the average size of Sm-AgNPs synthesized by *S. multicaulis* stem extract is 60 nm [7]. FE-SEM/EDX microanalysis was used to identify the composition of SmL-Ag-NPs and their relative abundance (Figure 3c). The EDX spectrum of SmL-Ag-NPs represents their pure elemental composition profile. As shown in the EDX spectrum, the most intense absorption peak observed at around 3 keV, which strongly verifies the presence of metallic silver (86.4%) as a major element [7]. Concurrently, the EDX spectrum revealed the presence of 6% of carbon, 4% of oxygen, and 3.7% of chlorine atoms in the formation of SmL-Ag-NPs (Figure 3c). The presence of gold ions in the EDX micrograph is due to the application of gold ions as cover during analysis. The absence of nitrogen ions in the EDX pattern indicates the complete reduction of Ag^+^ ions to Ag by bioactive plant metabolites [18]. Additionally, the presence of other elements in the EDX pattern confirms the tendency of plant-based phytochemicals in the reduction and stabilizing of silver ions during biosynthesis [4,7,18].

### 2.3. Size Distribution Analysis by Dynamic Light Scattering (DLS) Method

**Figure 4:**
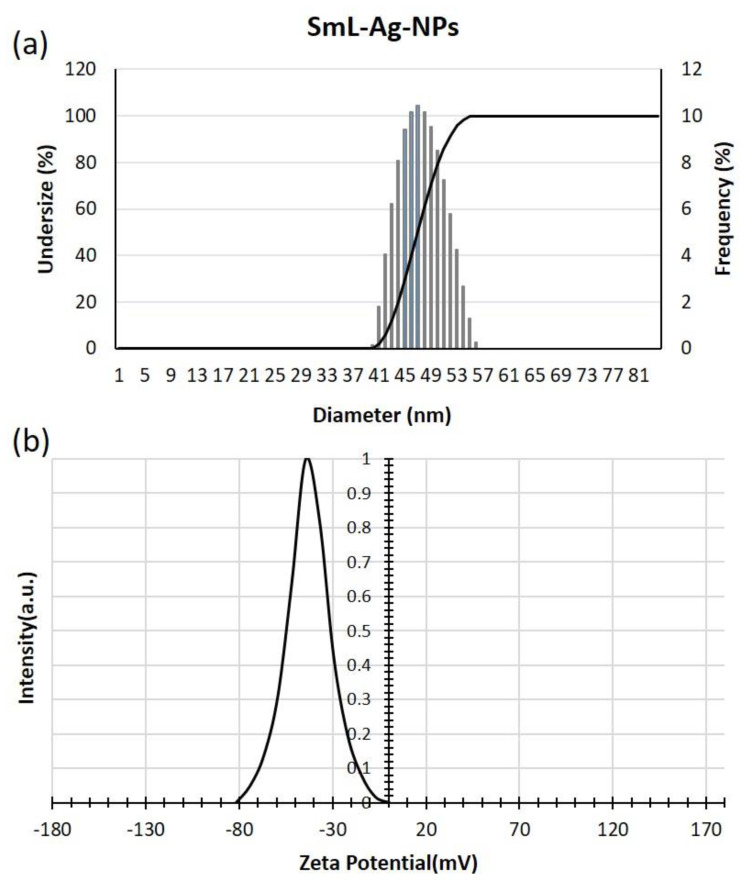
(**a**) DLS; and (**b**) zeta potential analysis of SmL-Ag-NPs. DLS: Dynamic light scattering.

DLS is an analytical tool most used to estimate the hydrodynamic diameter of biosynthesized nanoparticles. Size distribution analysis was performed to measure the particle size of biosynthesized SmL-Ag-NPs in an aqueous solution. It was determined that the average size of SmL-Ag-NPs was 46.9 nm with a Polydispersity Index (PDI) value of 0.112, which, based on the international standards organizations (ISOs) report, demonstrates its nano size, monodispersity, and homogeneity. Figure 4a illustrates that the particle size ranges from 41 to 56 nm.

Numerous studies have confirmed the difference in the size of silver nanoparticles synthesized by the green route obtained using the FE-SEM and DLS methods. Particle size analysis of biosynthesized silver nanoparticles using stem extract of *S. multicaulis* [7], *H. persicum* [18], and *C. intybus* leaf-derived callus [4] by the DLS system showed nanoparticles with sizes of 60, 42.9, and 36.1 nm, while using the FE-SEM method, the size of nanoparticles were approximately 40, 10, and 15 nm, respectively. Like previous studies, this research showed a larger size by the DLS technique than the microscopic test due to the thickness of the hydration shell caused by the aqueous environment of analysis.

### 2.4. Zeta Potential (ζ) Measurement of SmL-Ag-NPs

Zeta potential (ζ) is a physical parameter representing the surface charge potential of nanoparticles and a visible characteristic for their colloidal stability in aqueous suspensions, where nanoparticles with values between −10 and +10 mV are generally considered neutral, while nanoparticles with zeta potential values other than −30 mV to +30 mV are considered strongly anionic and strongly cationic, having better physical colloidal stability due to the electrostatic repulsion of individual particles.

As shown in Figure 4b, SmL-Ag-NPs were negatively charged with a high zeta potential of −42.6 mV, indicating good dispersion stability and increased electrostatic repulsion of SmL-Ag-NPs wrapped with anionic plant biomolecules in the colloidal suspension [7]. The presence of single hydroxyl groups (−OH) belonging to plant-origin phytochemicals trapped on the surfaces of SmL-Ag-NPs and their function as reducing and stabilizing agents is perhaps the leading cause of the anionic state of SmL-Ag-NPs [19]. Since the surface of most cellular membranes is negatively charged, these kinds of nanoparticles is less toxic in comparison to positively charged ones due to the electrostatic repulsion force between AgNPs and cells in the cytotoxicity effect [20].

### 2.5. Thermal Stability Analysis of SmL-Ag-NPs Using Thermogravimetric Analysis (TGA) and Derivative Thermogravimetry (DTG)

**Figure 5:**
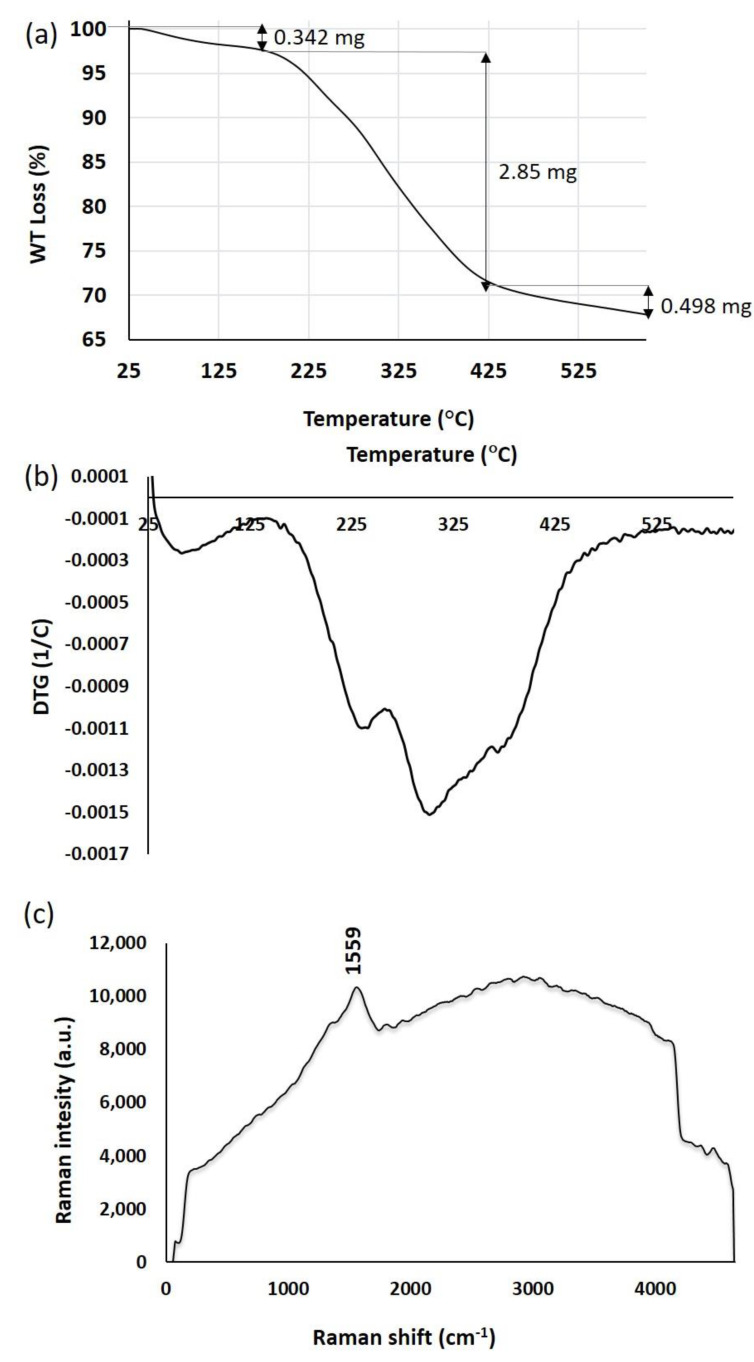
(**a**) TGA thermogram of SmL-Ag-NPs; (**b**) DTG curve of SmL-Ag-NPs; (**c**) Raman spectrum of SmL-Ag-NPs. TGA: Thermogravimetric Analysis; DTG: Derivative Thermogravimetry.

Biosynthesized SmL-Ag-NPs were analyzed for their thermal behavior by TGA and DTG. Figure 5a,b shows the TGA/DTG curves of the biosynthesized SmL-Ag-NPs. TGA is an effective analytical technique for measuring changes in the weight of a sample over time as the temperature changes during heating in a controlled atmosphere. DTG analysis is used to give a better insight into the thermal stability of materials. The SmL-Ag-NPs thermogram exhibited three visible decomposition stages at a temperature range between 25 °C and 610 °C at a rate of 10 °C per min under a nitrogen atmosphere (Figure 5a,b). The first step of weight loss (0.342 mg, 3%), which occurred below 190 °C, is possibly due to the desorption of volatile organic compounds and surface-adsorbed moisture. The second weight loss (2.85 mg, 24.96%) was between 190–425 °C, which is attributed to the decomposition of the plant-derived organic biomolecules such as flavonoids, phenolic acids, and carbohydrates trapped on the surface of SmL-Ag-NPs [7]. In the third stage, a steady weight loss (0.498 mG, 4.36%) was recorded between 425 °C and 610 °C, probably associated with the thermal degradation of oxygen molecules and resistant aromatic compounds present on the surface of SmL-Ag-NPs [21]. The TGA data indicated that the total decomposition of SmL-Ag-NPs due to the desorption of bioactive organic compounds was 32.32% (Figure 5a,b), illustrating the binding of bioactive and volatile organic compounds from the leaf extract to the surface of the obtained nanoparticles. A similar thermal behavior in green synthesized silver nanoparticles was observed for Sm-AgNPs synthesized using stem extract of *S. multicaulis* [7].

### 2.6. Surface-Enhanced Raman Scattering (SERS) Analysis of SmL-Ag-NPs

Raman spectroscopy is an analytical technique that is used to determine the chemical structure of metallic nanostructures and identify the possible functional groups of capping agents by measuring molecular vibrations. Figure 5c shows SERS spectra of SmL-Ag-NPs prepared using leaf extract of *S. multicaulis*. Raman spectroscopy of SmL-Ag-NPs did not provide any remarkable peaks. The only sharp band was the peak observed at 1574 cm^−1^, characteristic of silver nanoparticles [7]. In the previous study by Gharari et al. (2022), similar SERS spectra were recorded by AgNPs synthesized using stem extracts of *S. multicaulis* and *H. persicum* [7,18]. Liu et al. (2015) showed that noticeable Raman bands were observed in the samples containing dendritic, star-shaped hierarchical, and aggregated AgNPs compared to samples containing spherical silver particles [22]. The absence of remarkable peaks in SERS spectra of SmL-Ag-NPs could be due to the spherical structure of the nanoparticles.

### 2.7. X-ray Crystallography (XRD) Analysis of SmL-Ag-NPs

**Figure 6:**
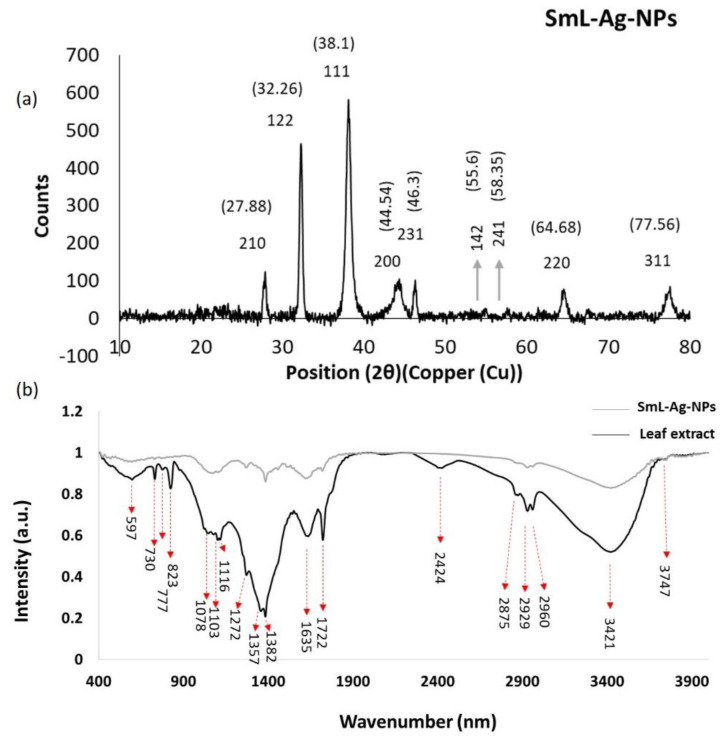
(**a**) XRD of SmL-Ag-NPs; (**b**) FTIR spectra in the comparative mode showing *Scutellaria multicaulis* leaf extract and biosynthesized SmL-Ag-NPs. XRD: X-ray diffraction analysis; FTIR: Fourier-transform infrared spectroscopy.

XRD analysis is a widely used technique to study the crystalline structure of materials to obtain information about their composition, structure, and physical properties. According to the JCPDS database, the Braggs peaks of SmL-Ag-NPs synthesized with *S. multicaulis* leaf extract at 2θ degrees are observed at 27.88°, 32.26°, 38.1°, 44.54°, 46.3°, 55.6°, 58.35°, 64.68°, and 77.56° (Figure 6a), which are matching to the 210, 122, 111, 231, 142, 241, 200, 220, and 311 planes for a standard sample of silver nanoparticles, respectively [4].

The XRD pattern of SmL-Ag-NPs clearly showed distinctive peaks corresponding to the crystal state of silver. Similar peaks have been reported from several AgNPs fabricated using plant extracts, and this result agrees with the report mentioned above [7]. Debye-Scherer’s equation D = Kλ/βcosθ was used to calculate the average size of SmL-Ag-NPs, where D is the crystallite size, K is the shape factor constant (0.9), λ (0.1540598 nm) is the wavelength of X-ray, β (0.01325019062) is the full width at half maximum in radian (FWHM), and θ (19.05 ≈ θ/2) is the Bragg’s angle. The average size of SmL-Ag-NPs is determined as approximately 11.07 nm from the breadth of the (111) reflection, which agrees with the size range provided by FE-SEM images (22–58 nm) and DLS analysis (41–56 nm). The XRD configuration of the SmL-Ag-NPs confirmed the formation of crystal structure nanoparticles, which govern their bioactivities and are favored by 111 facets.

### 2.8. Fourier Transform Infrared Spectroscopic (FTIR) Analysis

FTIR spectra of dried leaf powder extract of *S. multicaulis* and SmL-capped silver nanoparticles are shown in Figure 6b. FTIR is a powerful spectroscopy method for identifying the possible functional groups involved in the reduction of metal ions and the stabilization of synthesized nanoparticles. The FTIR spectrum of green synthesized SmL-Ag-NPs reveals clear absorption bands throughout the whole range of observation. FTIR analysis displayed visible bands at 3747, 3421, 2960, 2929, 2875, 2424, 1722, 1635, 1382, 1357, 1272, 1116, 1102, 1078, 823, 777, 730, and 597 cm^−1^ for synthesized SmL-Ag-NPs (Figure 6b). The band found at 3747 cm^−1^ can be attributed to hydrogen-bonded O–H stretching vibration [23]. The strong peaks at 3421 cm^−1^ correspond to the OH stretching of phenolic groups [24]. The absorption bands at around 2960 and 2929 cm^−1^ are related to –CH symmetric and asymmetric aldehydic C–H stretching vibrations, respectively [4]. The appearance of these peaks suggests the trapping of flavonoids and phenolic acids in the outer layer of the nanoparticles. The peak at 2875 cm^−1^ is attributed to the stretching vibrations of C–H groups [7]. The peak at 1722 cm^−1^ corresponds to the stretching vibration of the carboxyl carbonyl group [18]. The peak at 1635 cm^−1^ is assigned to stretching vibrations of the amide I arising from carbonyl stretch (C=O) in proteins [4]. The characteristic absorption peaks at 1382 and 1357 cm^−1^ correspond to the C–H bending vibration of the CH_3_ group or alkane [4]. The absorption near 1272 cm^−1^ would be assigned for C O groups [4]. Weak absorptions at 1116 cm^−1^, 1103 cm^−1^, and 1078 cm^−1^ are attributed to the C–O stretching alcohols [7]. The bands seen at 823 cm^−1^, 777 cm^−1^, 730 cm^−1^, and 597 cm^−1^ represent the aromatic groups of leaf extract that are involved in the reduction process of silver ions [7]. The shifting in wavenumber or changes in band intensity determine the types of functional groups that take part in the binding mechanisms [7]. The FTIR data indicate the participation of bioactive plant phytochemicals in both the synthesis and stabilization of SmL-Ag-NPs.

### 2.9. Phytochemical Composition Assay of SmL-Ag-NPs and S. multicaulis Leafs Extract

*S. multicaulis* stem extract was used as a reducing, capping, and stabilizing agent in the synthesis of Sm-AgNPs in our previous study [7]. Phytochemical analysis of *S. multicaulis* aqueous stem extract and Sm-AgNPs-free supernatant in negative ion mode using HPLC-MS^n^ analysis revealed the contribution of flavonoids such as jaceidin, skullcapflavon II, wogonin, oroxylin A, and dihydroxy and trimethoxy flavone in the formation of stable nanoparticles [7]. The potential medicinal properties of flavonoids trapped on the surface of biosynthesized nanoparticles lead to an increase in their biological activity [25]. There is a general belief that nanoparticles prepared from a specific plant extract or plant-derived biomaterial are likely to exhibit similar bioactivities to those exhibited by the plant extract [26]. Usually, nanoparticles derived from plant extracts show superior or better biological activity compared to plant extracts [27]. 

**Figure 7:**
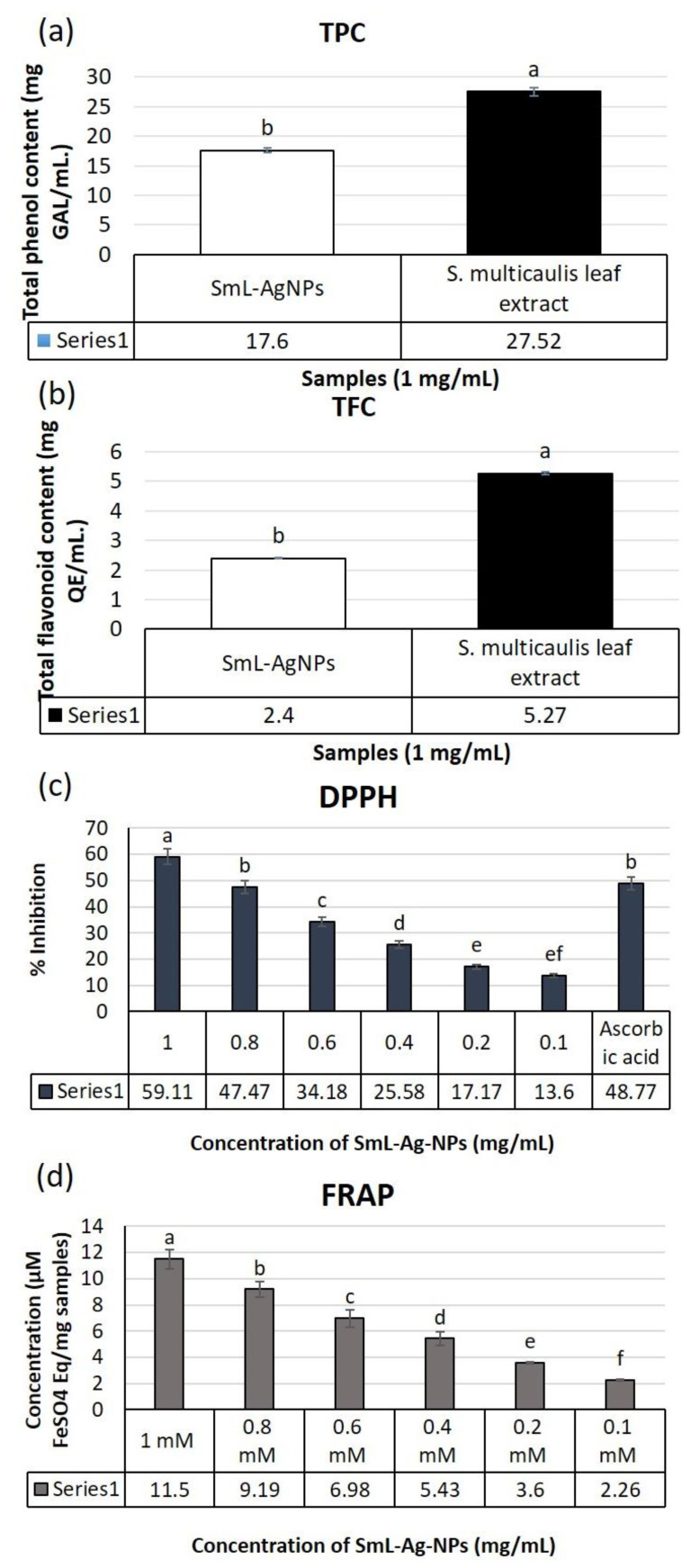
(**a**) Total phenolic content and (**b**) total flavonoid content of SmL-Ag-NPs and *S. multicaulis* leaf extract. Antioxidant activity of SmL-Ag-NPs by (**c**) DPPH and (**d**) FRAP assays. Different lower-case letters indicate a significant difference among different concentrations. DPPH: 2,2-Diphenyl-1-picrylhydrazyl; FRAP: Ferric Reducing Antioxidant Power Assay.

Since the *S. multicaulis* plant extract consists of a broad spectrum of oxygen-containing derivatives such as flavonoids, polyphenols, enzymes, sugars, and proteins, it could be employed as an excellent source of reducing and stabilizing agents for the extracellular formation of metal salts in metal nanoparticles. The TPC and TFC in the aqueous leaf extract and SmL-Ag-NPs were evaluated by the Folin–Ciocalteu and aluminum chloride colorimetric tests, respectively. A comparison between TPC and TFC of aqueous leaf extract and SmL-Ag-NPs showed that the TPC and TFC of aqueous leaf extract (27.52 ± 0.59 mg/G GAE and 5.27 + 0.33 mg QE/G extract) were higher than the TPC and TFC of SmL-Ag-NPs (17.6 ± 0.43 mg/G GAE and 2.4 ± 0.16 mg QE/G extract), (*p* < 0.01, Figure 7a,b). As certified by multiple studies, there is a strong correlation between plant total phenolic and flavonoid contents and its antioxidant potential [4,7,18]. Accordingly, the high contribution of phenolic compounds in reducing Ag ions could guarantee the wide range of bioactivities of SmL-Ag-NPs.

### 2.10. Antioxidant Activity of S. multicaulis Leaf Extract-Mediated Synthesized SmL-Ag-NPs

In the present study, the free radical scavenging activity of *S. multicaulis* leaf extract and of SmL-Ag-NPs synthesized by green synthesis was studied by using 2,2-Diphenyl-1-picrylhydrazyl (DPPH) free radical and Ferric Reducing Antioxidant Power Assay (FRAP) assay. DPPH is a very stable compound that can be reduced by accepting an electron or hydrogen from a hydrogen donor compound such as *S. multicaulis* leaf extract as an excellent source of various oxygen-containing secondary metabolites such as polyphenols, flavonoids, sugars, enzymes, and proteins [7]. A low IC_50_ value reflects the stronger scavenging activity of the plant extracts and plant-derived nanomaterials to act as DPPH scavengers, while a higher IC_50_ value indicates a lower antioxidant activity. The effect of various concentrations of SmL-Ag-NPs (0.1–1 mM) on DPPH radical antioxidant activity is shown in Figure 7c. Our results demonstrated that biosynthesized SmL-Ag-NPs are free radical scavengers, and the highest percentage of DPPH radical scavenging activity of SmL-Ag-NPs belonged to 1 mM with 59.11% inhibition. Furthermore, it was found that biosynthesized SmL-Ag-NPs had the lowest antioxidant activity at a concentration of 0.1 mM, with 13.6% inhibition (and with a mean IC_50_ value of 0.837 ± 0.09 mM) (Figure 7c). DPPH activity of SmL-Ag-NPs showed an increase in antioxidant activity in a dose-dependent manner ranging from 13.6% to 59.11%. At 1 mM of SmL-Ag-NPs and standard ascorbic acid, there was no significant difference in DPPH radical scavenging (Figure 7c).

In the FRAP assay, the ability of SmL-Ag-NPs to reduce Fe (III)-TPTZ to Fe (II)-TPTZ was significantly increased in a dose-dependent trend at concentrations ranging from 0.1–1 mM (Figure 7d). The highest reducing capacity belonged to 1 mM SmL-Ag-NPs with 11.5% inhibition. Statistical analysis proved that there was a significant difference (*p* ≤ 0.05) in the scavenging activity of the highest and lowest SmL-Ag-NPs concentrations (1 vs. 0.1 mM) (Figure 7d). There are some reports on the free radical scavenging activity of biosynthesized silver nanoparticles. Silver NPs’ synthesis, characterization, phytochemical content, antioxidant, and antibacterial and anticancer activities under in vitro conditions were investigated in *Heracleum persicum* stem extract [18], *Cichorium intybus* leaf-derived callus extract [4], *Scutellaria multicaulis* stem extract [7], and *Rhus coriaria* fruit extract [18]. The scavenging capacity of silver NPs evaluated by DPPH and FRAP assays differed due to different concentrations of AgNPs (100 to 600 μg/mL) [28]. Our results confirmed that *S. multicaulis* is a good source of natural antioxidants such as phenolic compounds and flavonoids. Phenolic and flavonoid compounds are important phytochemicals with an antioxidant capacity that is responsible for deactivating free radicals. SmL-Ag-NPs biosynthesized using leaf extract of *S. multicaulis* displayed scavenging activity due to trapped and capped phenolic compounds on their surface. Regarding earlier reports, there is a positive relationship between phenolic content and the antioxidant capacity of biosynthesized nanoparticles [4,7]. The radical scavenging activity of synthesized SmL-Ag-NPs is mainly related to the rich source of antioxidant phenolic compounds such as flavonoids (baicalein and wogonin and their glycosides, i.e., baicalin and wogonoside) in SmL-Ag-NPs which acted as stabilizing and capping agents.

### 2.11. Antibacterial Activity of SmL-Ag-NPs

**Figure 8:**
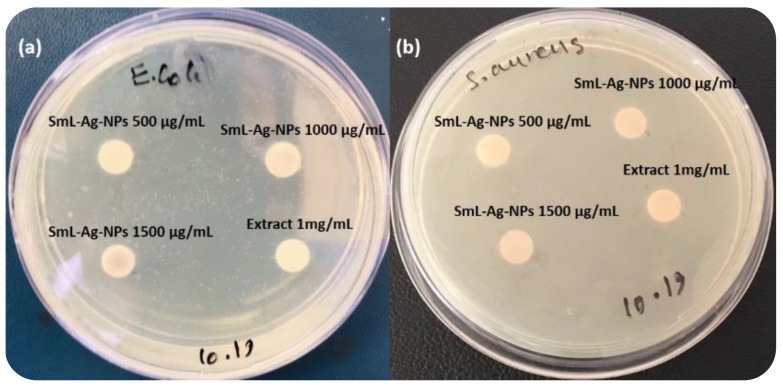
Antimicrobial assay of SmL-Ag-NPs against (**a**) *Escherichia coli* and (**b**) *Staphylococcus aureus* bacteria.

Although the specific mechanism of AgNP antimicrobial effects remains uncertain, it is proposed that AgNPs can continually release Ag ions, which may be considered a mechanism for killing microbes [29]. Owing to electrostatic attraction and affinity to sulfur proteins, Ag ions can adhere to the cell wall and cytoplasmic membrane. Adhered ions can enhance the permeability of the cytoplasmic membrane and lead to the disruption of the bacterial envelope [30]. After the uptake of free Ag ions into cells, respiratory enzymes can be deactivated, generating reactive oxygen species but interrupting adenosine triphosphate production. Reactive oxygen species can be a principal agent in provoking cell membrane disruption and deoxyribonucleic acid (DNA) modification. As sulfur and phosphorus are important components of DNA, the interaction of silver ions with the sulfur and phosphorus of DNA can cause problems in DNA replication and cell reproduction or even result in the termination of the microorganisms. Moreover, silver ions can inhibit the synthesis of proteins by denaturing ribosomes in the cytoplasm [31]. In the current study, the antimicrobial activity of SmL-Ag-NPs synthesized from *S. multicaulis* leaf extract was evaluated using the Kirby–Bauer Disk Diffusion method against Gram-positive *S. aureus* and Gram-negative *E. coli* bacteria. Water as a negative control and Nalidixic acid and streptomycin as positive control were considered. SmL-Ag-NPs showed no growth inhibition against either strain (Figure 8). The antibacterial activity of silver nanoparticles depends on different factors such as zeta potential, size, shape, colloidal state, temperature, pH, dose, and types of microbes [7]. Zeta potential is a physical feature of silver nanoparticles that controls their electrostatic interactions with bacterial surfaces. The resistance of bacterial strains towards SmL-Ag-NPs can be attributed to the fact that the surface of SmL-Ag-NPs is negatively charged, causing a lower tendency to electrostatic adhesion onto the bacterial surface [32]. On the one hand, due to the presence of negatively charged surface-acting agents on the SmL-Ag-NPs’ surface (zeta potential value of −42.6 mv) and, on the other hand, due to the presence of negatively charged carboxyl and amino groups belonging to the peptidoglycan layer in the bacterial cell wall, SmL-Ag-NPs did not display remarkable bactericidal effects towards bacterial strains. The results of this study are similar to the results of previous studies on the antibacterial effects of silver nanoparticles synthesized using stem extract of *S. multicaulis* (Sm-AgNPs) [7] and *H. persicum* (Hp-AgNPs) [18], which have the zeta potential values of −46.4 mv and −29.7 mv, respectively, without exerting antibacterial activity.

### 2.12. Cytotoxicity of SmL-Ag-NPs against MDA-MB231 Cells

**Figure 9:**
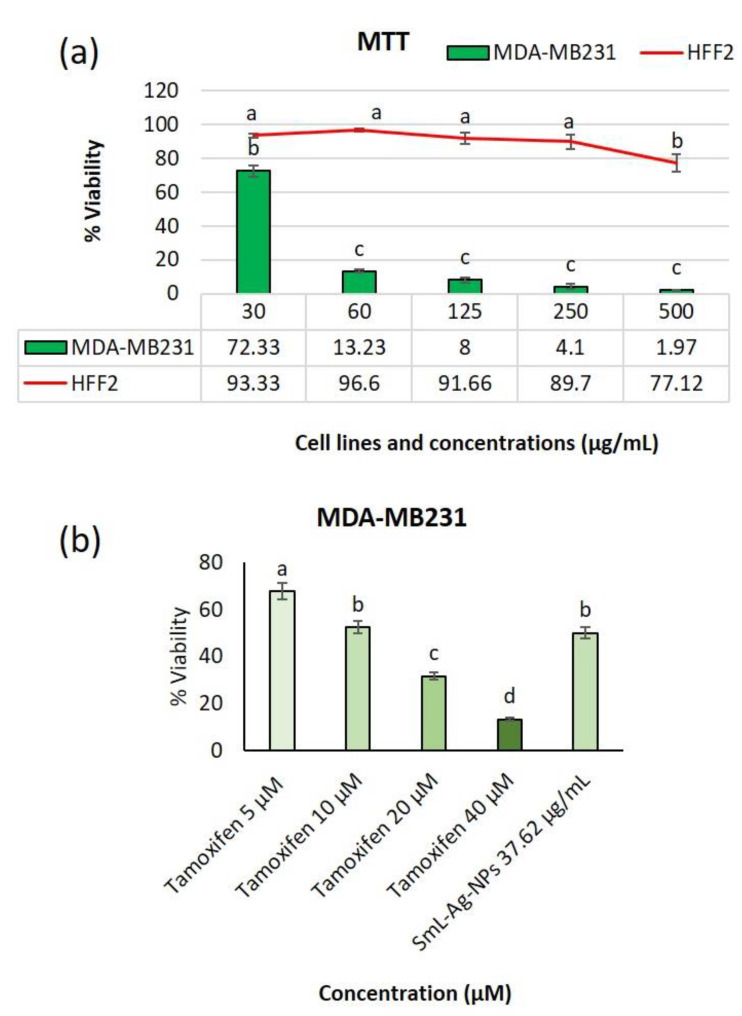
(**a**) Cytotoxicity of SmL-Ag-NPs on proliferation MDA-MB231 and HFF2 cells; (**b**) growth inhibition of MDA-MB231 cells by tamoxifen (5, 10, 20 and 40 µM) compared with SmL-Ag-NPs (37.62 μg/mL) after 48 h. Results are means ± SD (*n* = 3). Different lower-case letters indicate a significant difference among different treatments.

The anti-proliferative activities of the biosynthesized SmL-Ag-NPs were evaluated against the MDA-MB-231 human breast cancer cell line by MTT assay. Malignant MDA-MB-231 breast cancer cells and HFF-2 normal cells were incubated with various concentrations of SmL-Ag-NPs (30–500 μg/mL) for 48 h. Figure 8a shows the dose–response graph and calculated IC_50_ value of the MDA-MB231 cell line in the presence of SmL-Ag-NPs, after 48 h exposure. Our results revealed that SmL-Ag-NPs have higher toxicity properties against MDA-MB231 cells than HFF2 cells, which confirmed the selective cytotoxicity of SmL-Ag-NPs against tumor cells (Figure 9a). Based on the MTT assay, SmL-Ag-NPs displayed cytotoxicity towards MDA-MB231 cells in a dose-dependent manner with a low IC_50_ value of 37.62 μg/mL. Significant decreases in cell viability were observed at 125–500 μg/mL SmL-Ag-NPs, each 92%, 95.9%, and 98.03%, respectively (Figure 9a).

The anticancer activity of silver nanoparticles has been extensively investigated against the MDA-MB231 breast cancer cell line. In our previous study, Sm-AgNPs were synthesized with a diameter of 60 nm using *S. multicaulis* stem extract, and their cytotoxicity properties were evaluated on the MDA-MB231 and HFF2 cells [7]. The MTT assay showed that Sm-AgNPs decreased the cell proliferation rate at IC_50_ value at 81.2 μg/mL. Hanachi and colleagues synthesized Hp-AgNPs using *Heracleum persicum* stem extract, and their antitumor effect was tested on MDA-MB231 cells [18]. The IC_50_ value of these Hp-AgNPs was measured at 63.29 μg/mL at 48 h. In another study by Gharari et al. (2022), the anticancer activity of *Cichorium intybus* bio-callus synthesized silver nanoparticles (Ci-AgNPs) was investigated by MTT assay against MDA-MB231 cells. Cell viability was extensively decreased before Ci-AgNPs, and the IC_50_ value was calculated as 187.6 μg/mL at 48 h [4]. Compared with the literature, the current findings of the study showed that the green synthesized SmL-Ag-NPs had a significant inhibitory effect on the proliferation and growth of MDA-MB231 cancer cells at a lower IC_50_ value (37.62 μg/mL). Tamoxifen at four different doses (5, 10, 20, and 40 µM) was used as positive control (Figure 9b). MTT results showed that SmL-Ag-NPs have the potential to inhibit the multiplication of MDA-MB231 cells in a manner that is comparable with the tamoxifen (Figure 8b). Because of having leaky vessels, cancer cells mechanically facilitate the penetration of small-sized nanoparticles into these cells [33]. Morphologically, cancerous cells are characterized by having varying sizes and shapes as well as stable loss of cell-cell adhesion [34]. This suggests an increase in the disease-curing potential of nanoparticles by associating potentially bioactive peptides with various nanoparticles.

### 2.13. Apoptotic Effect of SmL-Ag-NPs on MDA-MB231 and HFF2 Cells

**Figure 10:**
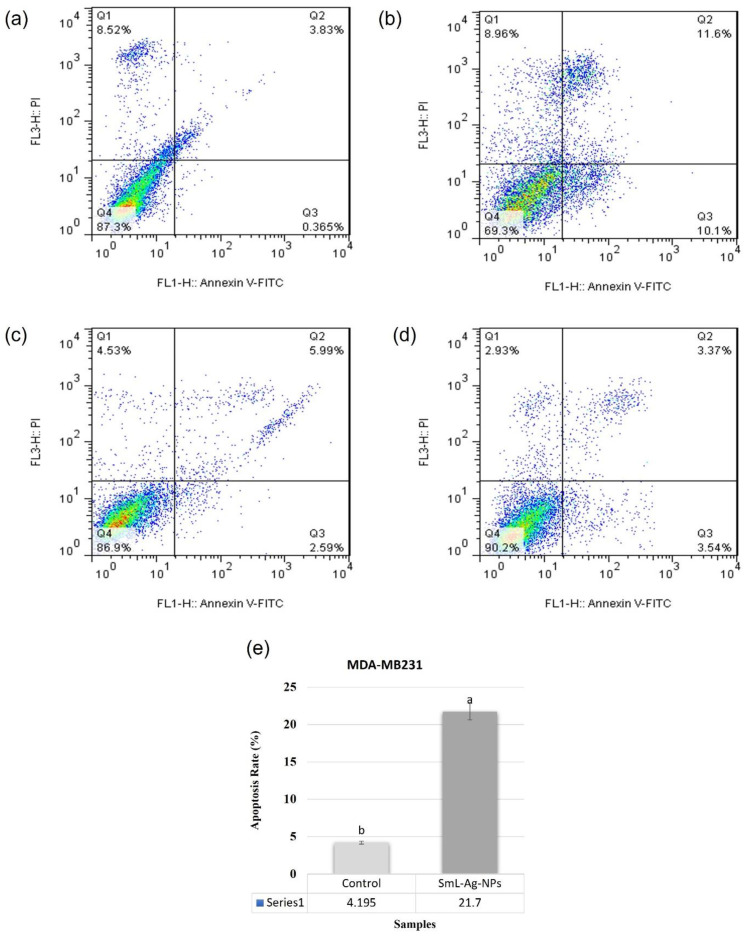
Apoptosis induced by SmL-Ag-NPs in MDA-MB231 cells was analyzed by flow cytometry using annexinV-FITC/PI kit (*p* < 0.01, *n* = 3). Representative dot plot showing viable cells (**lower left** quadrant), early apoptotic cells (**lower right** quadrant), late apoptotic cells (**upper right** quadrant), and necrotic cells (**upper left** quadrant) in (**a**) control MDA-MB231 cells, (**b**) MDA-MB231 cells treated with 37.62 µg/mL of SmL-Ag-NPs, (**c**) control HFF2 cells and (**d**) HFF2 cells treated with 37.62 µg/mL of SmL-Ag-NPs. Control refers to cells with no SmL-Ag-NPs treatment. (**e**) Apoptosis rate in untreated and treated MDA-MB231 cells. Different lower-case letters indicate a significant difference among different treatments. Figure 10a,c was reused with permission from Gharari et al. (2022), Analytical Biochemistry, https://doi.org/10.1016/j.ab.2022.114786.

The SmL-Ag-NPs-treated MDA-MB-231 cells were analyzed through flow cytometry by using PI and Annexin-V double staining to determine the apoptosis initiation and the cell’s percentage undergoing apoptosis. MDA-MB231 cells were treated with IC_50_ concentration of SmL-Ag-NPs (37.62 µg/mL) and were compared with tamoxifen and negative control after 48 h of incubation. SmL-Ag-NPs (37.62 µg/mL) treated MDA-MB-231 cells showed an increased number of cells undergoing early apoptosis, late apoptosis, and necrosis compared to control-treated cells. Figure 10a–d is a representative dot plot showing the percentage of untreated and SmL-Ag-NPs-treated MDA-MB231 and HFF2 cells undergoing early apoptosis (Annexin V-FITC^+^/PI^−^), late apoptosis (Annexin V^+^-FITC/PI^+^), and necrosis (Annexin-V-FITC^−^/PI^+^), respectively. As shown in Figure 10, the process of programmed cell death in the control groups (untreated MDA-MB231 and HFF2 cells) illustrated the natural course of these events during cell growth in culture. The percentage of cells undergoing early (10.1%) and late apoptosis (11.6%) was increased significantly (*p* < 0.01) in MDA-MB231 cells treated with 37.62 µg/mL SmL-Ag-NPs compared to that in the control cells (0.365% and 3.83%, respectively).

The population of early apoptotic MDA-MB231 cells increased from 0.365% to 10.1%, and the population of late apoptotic cells increased from 3.83 to 11.6% of the total cell population compared to untreated MDA-MB231 cells. Accordingly, the total percentage of apoptosis of the SmL-Ag-NPs-treated cells increased by about 17.5% compared to the control cells (Figure 10e), which verifies that SmL-Ag-NPs can induce selective apoptosis in cancer cells and lead to progressive apoptotic cell death. However, the number of cells undergoing necrosis increased slightly from 8.96% to 8.52% in treated cells compared to control cells. 

Although the silver nanoparticle mechanism of action in cell dysfunction and programmed death is unclear, their role in the generation of free radicals and induction of apoptosis has been reported by researchers [4,7,18]. Gharari et al. (2022) have reported the possible mechanism of apoptosis induced by Ci-AgNPs. Their results showed that *Cichorium intybus* bio-callus synthesized silver nanoparticles induce apoptosis by increasing the generation of reactive oxygen species (ROS) in mitochondria as a major source of ROS, followed by causing progressive oxidative damage in cellular compartments such as the endoreticulum, nucleus, and cell membrane [4]. 

A similar mechanism has been reported for silver nanoparticles synthesized using stem extract of *Scutellaria multicaulis* (Sm-AgNPs) and stem extract of *Heracleum persicum* (Hp-AgNPs) and their apoptotic effects [7,18]. Similar to these studies, our results propose that during the apoptosis process, SmL-Ag-NPs interact with the plasma membrane and trigger the production of free radicals and other reactive intermediates such as hydroxyl radical, hydrogen peroxide, superoxide, anion radical, hypochlorite, oxygen singlet, nitric oxide radical, and peroxynitrite radical, thereby leading to the severe structural and functional destruction of cellular biological macromolecules such as carbohydrates, lipids, proteins, and nucleic acids [35]. Increased production of free radicals with strong oxidizing abilities decreases the activity of the antioxidant systems consisting of superoxide dismutase (SOD), glutathione peroxidase (GPx), and catalase (CAT). Consequently, it results in lipid peroxidation and an increase in malondialdehyde (MDA), which leads to the development of oxidative stress and significant injuries to all biological molecules [36]. Gurunathan et al. (2013) concluded that synthesized silver nanoparticles using culture supernatant of *Bacillus funiculus* play a vital role in apoptotic mechanisms through the caspase-3 activation cascade [37]. Hsin et al. [38] also studied the apoptotic-induced cell death mediated by activating the mitochondrial JNP pathway in NIH3T3 fibroblast cells using nanosilver, which supports the current findings.

## 3. Materials and Methods

### 3.1. Collection of Plant Material and Preparation of Plant Extract

*S. multicaulis* leafs were collected from Tabriz, Iran. Taxonomic identification was approved by Dr. Amir-Hossein Talebpour (East Azerbaijan Research Center for Agriculture and Natural Resources, Tabriz, Iran). Dried leafs were ground by an electric grinder, and 5 g of the powdered sample was extracted in 100 mL of dH_2_O at 90 °C for 30 min in a mechanical shaker. The aqueous leaf extract was cooled at room temperature, and the solid residues were filtered through Whatman No. 1 filter paper and then centrifuged at 4500 rpm for 10 min. The supernatant was collected and maintained at 4 °C for the synthesis of SmL-Ag-NPs and biological assays [7].

### 3.2. Biosynthesis and Characterization of SmL-Ag-NPs Using S. multicaulis Leaf Extract

To synthesize SmL-Ag-NPs, 99, 97.5, and 95 mL AgNO_3_ solution (0.5, 1, and 2 mM) were mixed with 1, 2.5, and 5 mL of *S. multicaulis* aqueous leaf extract, respectively. The reaction mixture was sonicated for 30 min (three times) and then incubated overnight at room temperature under dark conditions to prevent the photo-activation of AgNO_3_ ions [39]. The formation of SmL-Ag-NPs was initially confirmed by the color change from a colorless to a brown-colored solution that then appeared dark brown after overnight incubation, which is caused by the bioreduction of Ag^+^ ions into Ag^0^ and the subsequent formation of SmL-Ag-NPs [4]. The preliminary characterization of SmL-Ag-NPs was confirmed by UV–Visible spectroscopy, and their absorbance was recorded in the range of 300 to 800 nm at a resolution of 1 nm. A final concentration of 2 mM AgNO_3_ was mixed with 5 mL *S. multicaulis* leaf extract as the optimum condition. The reaction mixture of SmL-Ag-NPs was centrifuged at 9000 rpm for 30 min to pellet the biosynthesized SmL-Ag-NPs. The pellet was rinsed three times with dH_2_O to remove impurities, excess unreacted Ag^+^ ions, and leaf extract residues and stored at 4 °C for assays.

### 3.3. Characterization of Biosynthesized SmL-Ag-NPs

UV-vis spectroscopy (Unico 2100) analysis was performed to record surface Plasmon resonance (SPR) peaks of biosynthesized SmL-Ag-NPs [18]. FE-SEM (FE-SEM ZEISS Sigma 300, Oberkochen, Germany) coupled with EDX was applied to observe the morphology and the corresponding elemental mapping of SmL-Ag-NPs [4]. A size analyzer (Vasco™ Flex, Cordouan Technologies™, Pessac, France) and Zetasizer instrument (SZ-100, HORIBA Scientific, Instrument Co., Ltd., Kyoto, Japan) were used to determine the size range and colloidal stability of SmL-Ag-NPs [7]. The thermal stability and weight loss of SmL-Ag-NPs were measured by thermo-gravimetric analysis (TGA) and derivative thermogravimetry (DTG) analysis [7]. The Surface-Enhanced Raman Spectroscopy (SERS) technique was used to record the SERS spectra of SmL-Ag-NPs to determine the functional groups involved in reducing SmL-Ag-NPs [18]. The crystalline nature of SmL-Ag-NPs was confirmed using an XRD instrument (X-ray diffractometer X’Pert Pro, Philips, Eindhoven, The Netherlands) [4]. To identify the plant extract-derived functional groups involved in the bioreduction of SmL-AG-NPs, FT-IR spectroscopy (FTIR Spectrum 2000, Perkin Elmer, 940 Winter Street. Waltham, MA 02451, USA) was performed. The spectra were recorded in the transmittance range of 400 cm^−1^ to 4000 cm^−1^.

### 3.4. Phytochemical Composition, Antioxidant Potential, and Antimicrobial Activity of SmL-Ag-NPs

The total phenolic content (TPC) and total flavonoid content (TFC) of SmL-Ag-NPs at different concentrations (e.g., 0.1, 0.2, 0.4, 0.8, and 1 mg/mL) and *S. multicaulis* leaf extract (1 mg/mL) were determined spectrophotometrically using the Folin–Ciocalteu reagent and aluminum chloride colorimetric methods, respectively [4]. The free radical scavenging potential of SmL-Ag-NPs (0.1–1 mg/mL) and *S. multicaulis* aqueous leaf extract (1 mg/mL) were measured using FRAP and DPPH assays [18]. The antibacterial activities of SmL-Ag-NPs (500, 1000, and 1500 μg/mL) and *S. multicaulis* aqueous leaf extract (1 mg/mL) were evaluated by using the disc diffusion method against *Escherichia coli* (ATCC 25922) and *Staphylococcus aureus* (ATCC29213). Streptomycin and Nalidixic acid (30 μg/disk) were applied to a positive control [4].

### 3.5. In Vitro Cell Viability Assay of MDA-MB231 and HFF2 Cells Treated with SmL-Ag-NPs

The cytotoxicity of SmL-Ag-NPs at different concentrations (30, 60, 125, 250, and 500 μg/mL) and *S. multicaulis* leaf extract at 1 mg/mL against the human breast carcinoma cell line (MDA-MB-231) and normal human foreskin fibroblast cells (HFF2) cells were determined according to the study by Gharari et al. (2022) and by using the conventional MTT-reduction test [7].

### 3.6. Cell Apoptotic Effect of SmL-Ag-NPs

Flow cytometric analysis was used to evaluate the apoptotic potential of SmL-Ag-NPs at IC_50_ concentration of 37.62 µg/mL in MDA-MB231 HFF2 cells after 48 h exposure using the Annexin V-FITC/PI staining kit following the manufacturer’s protocol [7].

### 3.7. Statistical Analysis

All experiments were performed in triplicate, and results were expressed as the mean of three repeats with standard deviation. The One-Way ANOVA procedure followed by Duncan’s new multiple-range test was used to determine the significance of the data. *p* values < 0.01 were highly statistically significant.

## 4. Conclusions

This research developed a rapid, simple, and eco-friendly protocol for the biosynthesis of nontoxic SmL-Ag-NPs. This was due to the higher content of phenolic phytochemicals with significant antioxidant activity against both DPPH and FRAP free radicals and potent and selective anti-proliferative properties against MDA-MB231 breast cells through cell growth inhibition and apoptotic effect, indicating their potential application as an anticancer remedial agent to overcome the drawbacks of current chemo drugs for breast cancer. However, further experimental studies are required to elucidate the exact underlying mechanism of SmL-Ag-NPs and their biological therapy for cancer in in vivo and in vitro models.

## Data Availability

The data presented in this study are available on request from the corresponding author.
