# Correction: Gharari et al. Eco-Friendly Green Synthesis and Characterization of Silver Nanoparticles by *Scutellaria multicaulis* Leaf Extract and Its Biological Activities. *Pharmaceuticals* 2023, *16*, 992

**DOI:** 10.3390/ph18101435

**Published:** 2025-09-25

**Authors:** Zahra Gharari, Parichehr Hanachi, Hanie Sadeghinia, Tony R. Walker

**Affiliations:** 1 Department of Biotechnology, Faculty of Biological Sciences, Alzahra University, Tehran 1993893973, Iran; sadeghinia.h@yahoo.com; 2 School for Resource and Environmental Studies, Dalhousie University, Halifax, NS B3H 4R2, Canada; trwalker@dal.ca

**Error in Figure and Legend**

In the original publication [1], there was a mistake in Figure 10 as published. Figure 10c,d was mistakenly swapped during pasting for paper preparation. There was also a mistake in the legend for Figure 10. The caption for Figure 10 incorrectly assigns Figure 10c as the control for non-treated HFF2 cells when it actually depicts HFF2 cells treated with SmL-Ag-NPs, while Figure 10d correctly shows untreated HFF2 cells. The corrected Figure 10 and caption appears below. The authors state that the scientific conclusions are unaffected. This correction was approved by the Academic Editor. The original publication has also been updated.

## Figures and Tables

**Figure 10:**
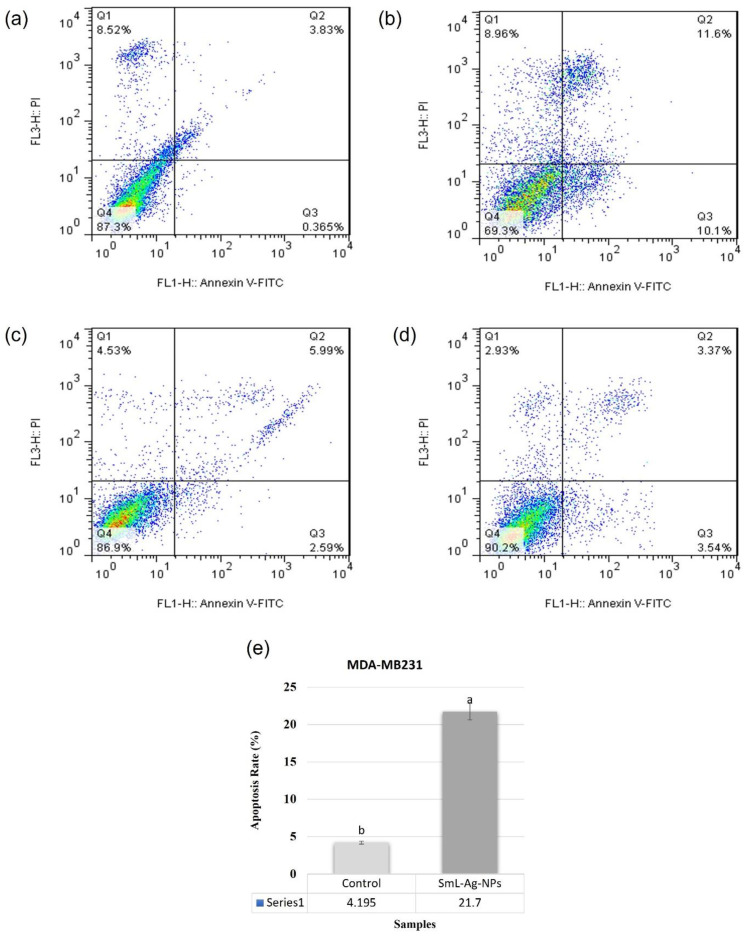
Apoptosis induced by SmL-Ag-NPs in MDA-MB231 cells was analyzed by flow cytometry using annexinV-FITC/PI kit (*p* < 0.01, *n* = 3). Representative dot plot showing viable cells (**lower left** quadrant), early apoptotic cells (**lower right** quadrant), late apoptotic cells (**upper right** quadrant), and necrotic cells (**upper left** quadrant) in (**a**) control MDA-MB231 cells, (**b**) MDA-MB231 cells treated with 37.62 µg/mL of SmL-Ag-NPs, (**c**) control HFF2 cells and (**d**) HFF2 cells treated with 37.62 µg/mL of SmL-Ag-NPs. Control refers to cells with no SmL-Ag-NPs treatment. (**e**) Apoptosis rate in untreated and treated MDA-MB231 cells. Different lower-case letters indicate a significant difference among different treatments. Figure 10a,c was reused with permission from Gharari et al. (2022), Analytical Biochemistry, https://doi.org/10.1016/j.ab.2022.114786.
